# Stakeholder Perspectives on Identifying and Managing Comorbid Health Conditions Among Nursing Home Residents with Dementia: A UK Single-Site Qualitative Study

**DOI:** 10.3390/healthcare14162601

**Published:** 2026-08-18

**Authors:** Hannah Lancaster, Frances Hawes, Eugene Y. H. Tang, Serena Sabatini

**Affiliations:** 1 School of Psychology, Faculty of Health and Medical Sciences, University of Surrey, Guildford GU2 7XH, UK; hannahyvettelancaster@gmail.com; 2 Health Care Administration Department, University of Wisconsin Eau Claire, Wisconsin, WI 54071, USA; hawesfm@uwec.edu; 3 Population Health Sciences Institute, Newcastle University, Newcastle NE4 5PL, UK; eugene.tang@newcastle.ac.uk; 4 Department of Clinical Psychology and Psychobiology, University of Barcelona, Campus Mundet, Ponent, Passeig de la Vall d’Hebron, 171, 08035 Barcelona, Spain; 5 Institute of Neurosciences, University of Barcelona, 08035 Barcelona, Spain

**Keywords:** nursing home, institutionalization, co-morbidity, Alzheimer’s, qualitative interviews, informal caregivers, care staff, nurses, general practitioner

## Abstract

**Background:** This single-site qualitative interview study sought the opinions of 15 stakeholders from a nursing home based in the United Kingdom (UK) about identification and management of comorbidities in residents with dementia. **Methods:** Stakeholders were recruited through one of the nursing home owners, who acted as the gatekeeper. No residents with dementia were interviewed. Semi structured interviews were conducted online via Microsoft Teams in 2024. Data was analyzed using thematic analysis. **Results:** Stakeholders comprised four care assistants; three well-being companions; two nurses; a general practitioner (GP); two informal caregivers; a chaplain; the manager; and the owner of the nursing home. Stakeholders perceived that the identification and management of comorbidities in residents with dementia were shaped by interacting factors across four domains: the wider healthcare context, nursing home organization, staff capability and care practices, and resident-related challenges. Perceived healthcare under-resourcing increased complexity, while coordinated teamwork, continuous improvement, staff well-being, geriatric expertise, and person-centred, inclusive care were viewed as key facilitators. **Conclusions:** Overall, the perspectives of stakeholders interviewed in this single-site case study suggest that improving the identification and management of comorbidities in residents with dementia requires multilevel interventions that strengthen staff capability, organizational practices, and integration between nursing homes and the wider healthcare system.

## 1. Introduction

Globally, 55 million people have dementia, including approximately 994,000 in the United Kingdom (UK) [1]. Dementia encompasses various neurological conditions characterized by a significant decline in cognitive functions, such as memory and language, that interferes with individuals’ ability to conduct everyday activities [2]. Moreover, most people with dementia experience behavioral and psychological symptoms such as depression [3]. In the UK, about 39% of people with dementia live in residential care [4]. Residential care settings encompass several types of long-term care facilities, including residential care homes, nursing homes, dementia care homes, assisted living, sheltered housing, continuing care retirement communities, and palliative care homes. Residential care homes provide accommodation, personal care, meals, social activities, and nursing care, whereas nursing homes additionally support residents with greater functional impairments and activities of daily living. Dementia care homes offer secure environments and staff trained in dementia care. Assisted living and sheltered housing promote independent living while providing varying levels of on-site support. Continuing care retirement communities offer a continuum of care, enabling residents to transition as their needs change, whereas palliative care homes provide end-of-life care focused on comfort and quality of life.

Although epidemiological data on UK residential care settings is limited, around 90% of community-dwelling individuals with dementia have at least one comorbid condition (e.g., diabetes) and one or more health issues (e.g., swallowing issues, recurrent falls). For this study, a comorbid condition was defined as a diagnosed or suspected health condition occurring alongside dementia. Interaction between its symptoms and dementia was considered clinically relevant but was not required for classification. Existing evidence documents cardiovascular diseases, diabetes, movement disorders, chronic infections, and depression as the most common comorbid conditions to dementia [5,6]. Since these conditions are chronic, they persist and often worsen when individuals transition to residential care. Consequently, these conditions are common among people with dementia living in residential care. Additionally, people with dementia will likely develop new health conditions while living in residential care [7,8]. Hence, most nursing home residents with dementia have multiple health needs.

The importance of paying attention both in research and clinical practice to the overall health state of people with dementia has been underlined in the 2024 Lancet commission on dementia prevention, intervention, and care [9]. Early identification and management of comorbidities is crucial, as multiple comorbidities increase the risk of faster cognitive decline, functional deterioration, and earlier mortality in people with dementia [6,10,11]. Conversely, advancements on detection and management of dementia comorbidities could help avoid unnecessary hospitalizations and reduce healthcare costs [9]. Better management of dementia comorbidities in residential care would also improve quality of life for residents, families, and staff.

Research suggests that nursing home residents with dementia and comorbidities receive different treatments than people with the same condition but without dementia [12]. Moreover, communication and behavioral difficulties may limit medical examinations in people with dementia, decreasing the likelihood of receiving new diagnoses [13,14]. Additionally, concerns about side effects and drug interactions may lead to less medication use in people with dementia [15,16,17]. Nonetheless, disparities in the treatment of people with and without dementia in residential care have not been consistently reported [18]. Moreover, the available quantitative evidence from residential care settings has documented the challenges of dealing with a few specific comorbidities (e.g., Parkinson’s disease) and health issues (e.g., pain) in dementia. A study found that despite 7.4% of nursing home residents having Parkinson’s disease and dementia, staff did not have sufficient knowledge of these conditions [19]. Another study found that nurses understood pain in dementia but lacked clear guidelines for its detection and management [20]. The challenges related to other common comorbidities to dementia have received less attention.

Qualitative studies are key to advance understanding on how nursing home staff perceive and manage comorbidities in residents with dementia. However, previous evidence has largely examined nursing home staff’s experiences of dementia care in general (e.g., barriers to person-centered care, management of behavioral and psychological symptoms, and staff training needs); [21,22,23], rather than on the specific challenges associated with identification and management of comorbidities in residents with dementia. Understanding these challenges is important because the management of comorbidities requires staff to balance dementia-related needs with the assessment and treatment of other chronic conditions, which may complicate clinical decision-making and influence residents’ quality of care.

To the best of our knowledge, only two qualitative studies specifically focused on the management of comorbidities in nursing home residents with dementia. The first is a study conducted in the United States (US) and exploring the challenges of caring for residents with dementia and comorbidities using open-ended survey questions completed by 38 nursing home staff from nine nursing homes [24]. Staff identified dementia-related symptoms, limited training, staff shortages, healthcare-system barriers, and communication with residents’ families as key challenges [24]. The second is an expert consensus study conducted in the UK [25]. Similarly to what was previously reported [24], stakeholders in this study also considered limited training and staffing shortages as challenges that negatively impact management of comorbidities in nursing home residents with dementia. Additional important challenges reported in this study were limited access to General Practitioners (GPs) and other external specialists, leading to long waiting times during medical emergencies. Although these studies identified several important barriers to managing comorbidities among nursing home residents with dementia, they provide limited insight into how these challenges arise and are addressed in routine practice. The use of open-ended survey questions limited the depth of participants’ responses in one of these studies [24], while in the other [25] the Delphi methodology was designed to achieve expert consensus rather than explore stakeholders’ lived experiences.

Overall, little is known about how organizational and staff factors interact in the day-to-day management of comorbidities within nursing homes. A single-site qualitative interview study offers the opportunity to generate rich, contextualized accounts of these processes within a real-world care setting. This single-site qualitative interview study therefore gathers the perspectives of various stakeholders involved in the care of residents with dementia in a UK nursing home to explore the challenges (aim 1) and facilitating factors (aim 2) associated with identifying and managing comorbid conditions in residents with dementia.

## 2. Materials and Methods

### 2.1. Study Design

An interview-based qualitative case study design was used to gather the stakeholders’ perspectives [26,27]. A case study can be defined as “an in-depth exploration from multiple perspectives of the complexity and uniqueness of a particular project, policy, institution, program or system in a ‘real life’ context” [28], p. 21. In this study, a “case” was defined as a nursing home facility. A nursing home was selected as it is where people with dementia often live due to severe functional limitations. A single-site case study approach was chosen because we were interested to collect the opinions of different types of professionals exposed to the same care environment to avoid variability caused by institutional differences.

### 2.2. Data Collection

Recruitment and data collection occurred between May and October 2024. The inclusion criteria for the nursing home to participate in the study were to provide care to residents with dementia and comorbidities and to be located in the UK. To identify nursing homes to invite to participate in the study researchers referred to the list of nursing homes from the Care Quality Commission website. Fourteen nursing home owners were contacted by email, and two agreed to participate but only one was eligible as the other was a care home. Hence, a convenience sample was selected. Fifteen nursing home staff and informal caregivers were recruited through one of the nursing home owners who acted as gatekeeper (the selected nursing home has more than one owner). All invited staff members (i.e., nurses, care assistants, well-being companions, manager, chaplain, and GP) voluntarily decided to participate, whereas two out of three informal caregivers agreed to participate. The chaplain was included as an additional stakeholder because of their regular contact with residents, families, and staff. Although not directly involved in clinical care, the chaplain provides a holistic perspective on residents’ well-being and may observe changes in residents’ physical, emotional, and psychosocial functioning that are relevant to the identification and management of comorbidities.

Based on the availability of the nursing home owner, two days were selected to undertake interviews with staff members, whereas the GP, chaplain, and informal caregivers were all interviewed on separate dates. Staff members who were doing their shift during the interview days were invited to take part in the research, and their decision to participate was voluntary. Participants read the information sheet and signed a digital consent form via email. Data was collected through videorecorded online interviews conducted via Microsoft Teams. Participation to the study was not remunerated. The study obtained favorable ethical approval from the Ethics Committee of the University of Surrey (EGA ref: FHMS 23-24 092 EGA).

### 2.3. Description of the Nursing Home

The participating nursing home was a family-run facility located in the UK, housing up to 47 residents. At the time of data collection, the nursing home housed 36 residents, including those with a diagnosis of dementia and those without dementia. Residents’ ages ranged between 80 and 103 years old. Among residents, 19 had a diagnosis of dementia but, according to the GP, many more had probable dementia even though they did not receive a formal diagnosis. Additionally, the GP reported that levels of dementia and functional limitations varied among residents (from mild to severe dementia), even though the nursing home is advertised as providing care only to individuals with mild to moderate levels of dementia. One of the nursing home owners reported that common comorbidities and health issues among residents with dementia are high blood pressure and coronary diseases, arthritis, osteoporosis, falls and fractures, poor mobility, diabetes, chronic obstructive pulmonary disease, chest infections, incontinence, urinary tract infections, kidney disease, hearing and vision loss, depression, anxiety, pressure sores, poor nutrition, weight loss, and swallowing problems. The investigated nursing home is not a dementia-specific home and therefore it does not follow a specific dementia-care model, but it reports adhering to a more general person-centered approach. Moreover, the nursing home has obtained several prizes for the quality of its care. Finally, the investigated nursing home adopts a predominantly self-funded (privately paid) model. In fact, in the investigated nursing home 95% of residents were self-funded. In the UK, residential care may be financed through private payment, local-authority means-tested funding, or NHS funding. Unlike local-authority funding, NHS funding is based on assessed healthcare needs rather than an individual’s financial circumstances.

Adjacent to the nursing home, the same owners operate a set of independent living apartments. To facilitate residents transition from independent living to the nursing home within the same complex as their care needs increase. The nursing home complex also offers daily respite care for community-dwelling older adults, and it is equipped with the facilities and professionals to provide palliative and end-of-life care, including provision of spiritual/religious support.

The nursing home has been privately managed for 20 years. The owners oversee a team that includes the nursing home manager, 12 nurses, 13 care assistants, four well-being companions, four kitchen staff, two administrative personnel, and four housekeeping staff members, all working daily. According to one of the nursing home owners, in the investigated nursing home, staff members worked on average 6.2 years. More specifically, nurses worked on average 5.6 years and care assistants worked on average 2.7 years. The nursing home does not have a fixed staff-to-resident ratio. However, one of the nursing home owners said that there are always two nurses on shift during the day and night. Moreover, there are nine care staff on shift during the day and four during the night. In addition, there are two well-being specialists on duty from Monday to Friday. Also, the nursing home has a long-standing agreement with a GP surgery, which consists of a weekly three-hour GP visit within the nursing home and remote online support in exchange for a fee paid by the nursing home. This occurs in a UK context where GP surgeries are commissioned to provide care to local nursing homes, but the frequency of visits is determined locally [29]. Additionally, for residents who (or whose family) request it, the nursing home has arrangements with a physiotherapist who provides on-site services for an extra fee paid by residents’ families. A chaplain is also employed by the nursing home to meet with residents twice a week and to deliver a service once a month. The employed nurses have varying degrees of specialization, including clinical lead nurses, senior registered nurses, and registered nurses. In the UK, registered nurses provide direct care, manage medications, investigate health issues, and liaise with GPs and other specialists. Clinical lead nurses oversee care and support residents, families, and staff, while care assistants provide hygiene-related care. Finally, the well-being companions were responsible for delivering group-based and one-on-one activities. For an additional fee paid by residents’ families, these specialists could accompany nursing home residents to various places, including medical appointments at hospitals, pharmacies, and retail stores. Overall, the selected nursing home may represent a positive-deviant case in the UK landscape.

### 2.4. Interviews

Interviews lasted between 20 and 70 min, with an average of 45 min. All interviews were jointly conducted by two researchers: a psychologist and researcher with expertise in dementia and aging, and a master’s student in Psychology with knowledge of dementia. Interviews occurred during working hours for the nursing home manager, nurses, care assistants and well-being specialists. Staff were reassured their participation was voluntary. Moreover, interviews were conducted in a private and isolated room to enable participants to speak freely, and participants were reminded that the videorecording of their interviews would have been deleted after transcription and that the transcriptions of their interviews would have been made anonymous. Except for the informal caregivers, during the interviews the remaining stakeholders were first asked to describe their role, their education, and prior work experiences. Successively, they were asked which dementia comorbidities were most difficult to manage, the protocols and training in place to deal with such comorbidities, and how care could be improved for people with dementia with comorbidities. Primary informal caregivers were asked about residents’ dementia history, cognitive and functional status, comorbidities, admission to the nursing home, interactions with staff, and involvement in care planning. Before starting the survey, participants were informed that comorbidities referred to one or more medical conditions in addition to dementia, whose symptoms may interfere with those of dementia. The full interview script can be found in Supplementary Text S1. As it can be noted in the interview script, interviews only focused on management of comorbid conditions in nursing home residents with dementia. However, many stakeholders mentioned aspects related to identification of comorbid conditions among residents with dementia. Consequently, both identification and management of comorbid conditions among nursing home residents with dementia became the focus of this study.

### 2.5. Data Analyses

**Figure 1:**
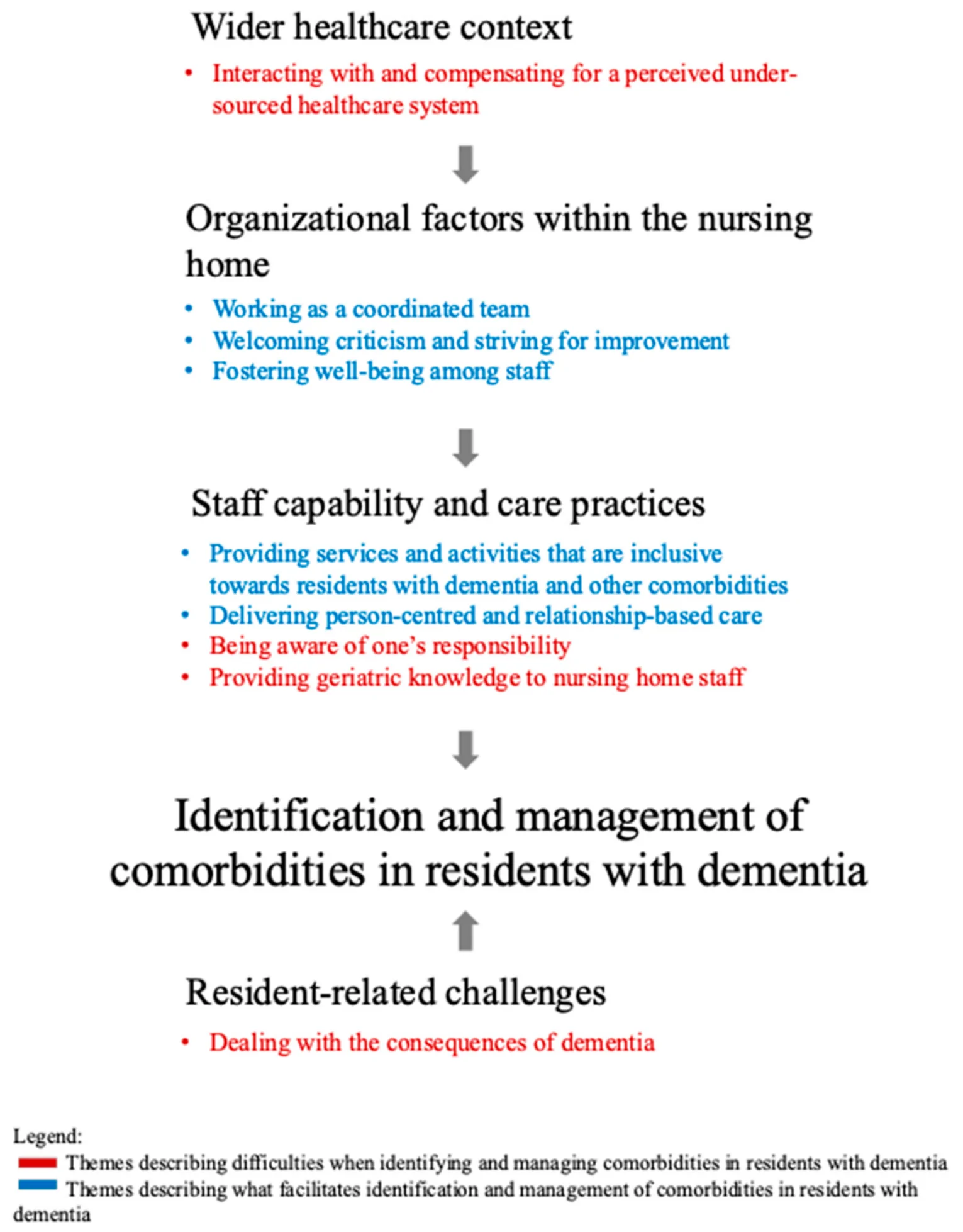
Conceptual model of stakeholders’ perceived factors influencing the identification and management of comorbidities in nursing home residents with dementia.

Primary data analysis was conducted. The video-recorded interviews were transcribed by two researchers (HL and SS), yielding to 211 pages of transcripts, with each interview covering stakeholders’ opinions regarding identification and management of comorbidities in nursing home residents with dementia. The length of transcripts ranged from 10 pages to 26 pages. Two analyses were conducted, one for each study aim, and two researchers (HL and SS) independently undertook both analyses. Transcripts were thematically analyzed using a constant comparative method approach [30,31,32] which allows for iterative comparing of emergent concepts to identify patterns in the population’s experiences (i.e., themes). Two researchers (HL and SS) independently conducted codebook thematic analysis after immersing themselves in the data (re-reading of transcripts several times), keeping analytic memos. Using the analytical steps outlined by Strauss and Corbin (1998) [32], they sought to: (1) identify concepts with the text and assigning them labels (i.e., codes), (2) collapse these codes into themes and subthemes [33], and (3) provide a definition for each theme and subtheme, ensuring a rich description [34]. A codebook was developed and continuously revised through frequent meetings to establish and define themes and subthemes and their definitions. Disagreements between the two coders were handled with open discussion between researchers via video meetings. Coding labels, themes, and subthemes were then redefined and corroborated between researchers to ensure consensus. Finally, a conceptual model (Figure 1) was produced to illustrate the identified themes and subthemes. The conceptual model integrated results from the two codebook thematic analyses. Findings are presented primarily through analytic summaries supported by selected illustrative excerpts. Data were managed and coded using Delve, a cloud-based qualitative data analysis software.

Trustworthiness of the analysis was supported through several strategies. First, credibility was enhanced using semi-structured interviews, audio-recording and verbatim transcription of interviews, regular online discussions among the research team throughout the analytic process, and the inclusion of illustrative excerpts to support the interpretation of the findings. Second, dependability was promoted through the development of an iterative codebook and independent coding of the data by two researchers, with discrepancies discussed until consensus was reached. Reflexivity was supported through regular discussions within the multidisciplinary research team to consider how researchers’ disciplinary backgrounds, prior experiences, and assumptions might influence data interpretation. In this regard, neither researcher had previous professional or personal relationships with the selected nursing home or stakeholders prior to the study. While both researchers brought an interest in improving the care and well-being of people living with dementia, neither had a professional background in nursing home practice. Throughout data collection and analysis, the researchers remained aware that their disciplinary backgrounds and perspectives could shape the interpretation of participants’ accounts and sought to maintain an open and exploratory approach to the data, allowing themes to emerge inductively from participants’ experiences.

## 3. Results

### 3.1. Participants

There were 15 participants: four care assistants; three well-being companions; two nurses; the manager (administrator); one of the nursing home owners; the GP assigned to the nursing home; two informal caregivers of residents; and a chaplain. Table 1 reports participants’ gender, their roles, years of experience in the nursing home and care sector, their professional qualifications, whether they undertook training in dementia care, and their employment status. Among participants, nurses worked on average 8.5 years, care assistants worked on average 3.5 years, and well-being companions worked on average 2.7 years. To provide some context, in the UK 34.9% of nursing home staff resign from their job each year [35]. Relatives of the two informal caregivers had been living in the nursing home complex for 16 and 8 years, respectively. Both residents initially lived in independent living apartments before moving to the nursing home when they required nursing care.

### 3.2. First Study Aim

The following five themes address the perceptions of stakeholders of a UK-based nursing home with regard to difficulties related to identification and management of comorbid conditions in nursing home residents with dementia: dealing with the consequences of dementia; providing geriatric knowledge to nursing home staff; providing services and activities that are inclusive towards residents with dementia; interacting with and compensating for a perceived under-sourced healthcare system; and being aware of one’s responsibility.

### 3.3. Dealing with the Consequences of Dementia

Identifying and managing additional health issues in people experiencing the core (e.g., memory loss) and the behavioral and psychological (e.g., agitation) symptoms of dementia was perceived as the biggest challenge. This theme comprised four subthemes: ensuring day-to-day care to residents with memory impairment; interacting with residents with limited communication; ensuring safety in residents who may not be aware of their impairments; and dealing with the behavioral issues of dementia.

**Ensuring Day-to-Day Care to Residents With Memory Impairment.** Stakeholders reported that residents with dementia may forget not only the social activities they have booked or the meals they have ordered, but also to wear their glasses or hearing aids, attend scheduled medical appointments, or take their medications. As a result, meeting both their psychological and physical care needs becomes challenging and requires tailored care strategies. For example, to reduce the risk of residents forgetting to take medications an informal caregiver explained how they got advised to take all medications together once or twice a day: *We get the dosages [of medications] down to once a day or twice a day maximum to increase compliance because the more often doses are the more time someone has to self-medicate during a day, the more risk of them [residents with dementia] failing.* Moreover, stakeholders reported that due to forgetfulness and confusion about the identity of medical specialists, residents may get agitated and refuse to participate in scheduled medical visits which delays recognition of new health conditions, as well as management of comorbid health issues.

**Interacting With Residents With Limited Communication.** Care assistants and nurses reported that residents with severely impaired communication cannot express pain and physical discomfort to them, making it difficult to promptly recognize new health conditions and, consequently, to prevent complications. For example, they may be unable to communicate when they are constipated and, thus, they risk being hospitalized for something that could have been prevented. About this, a nurse said: *Because of the dementia, they [residents] can’t really always verbalize if they’ve had their bowels open or not and sometimes, it’s hard to keep track of their bowels. And we’ve had situations where people have forgotten how many days it’s been since they last opened their bowels and they’ve had like problems with being admitted to hospital only after we realized that the medical history showed that they had bowel obstructions.* According to stakeholders, residents with dementia also tend to have difficulties communicating with specialists during medical visits, and especially during online consultations. To compensate for the communication difficulties of residents with more advanced dementia, care assistants, nurses, and the GP, all reported having to adopt an inquisitive attitude, to focus on bodily cues and mood changes, and to make the best guess about the new health issues the residents are facing. For example, a care assistant stated: *They [residents with dementia] cannot express their feelings. We have to understand their expressions.* In this regard, care assistants explained that when they suspect a new health issue, they report it to the nurses, who in turn inform the GP who will ultimately undertake a medical assessment and propose medical decisions.

**Ensuring Safety to Residents Who May Not be Aware of Their Impairments.** According to stakeholders, residents with dementia may engage in risky behaviors that can exacerbate the conditions they have and even lead to new health issues. For example, residents with mobility issues may continue walking unattended or without using a walking aid and fall. This led to a feeling of frustration resulting from not being able to prevent those new health issues that are caused by residents’ risky behaviors. In this regard, a care assistant reported: *I feel something much difficult is they forget to ask for help and try to do things by themselves, and they finally may fall down, or they may get bruises or something. So that’s the thing that I feel they forget to ask for help and try to do things by themselves*.

**Dealing With the Behavioral Issues of Dementia.** According to participants, behavioral issues among residents with dementia were common and required staff to continually adapt their approaches because of their changing nature. To emphasize this, a care assistant highlighted: *It is challenging because they [behavioral issues] are not the same everyday.* Stakeholders explained that when poorly managed, issues such as agitation and depression can impede care-related tasks and medical visits, leading to delayed identification and management of comorbid health issues. However, at the same time, stakeholders explained that in some cases medications against behavioral issues may not be suitable for people with dementia with other comorbidities. In these cases, management of the comorbid condition may be prioritized over treatment for behavioral issues. For example, the GP said: *Behavior is probably the biggest aspect and it’s the most difficult to treat because even when it comes to medication, sometimes medications that we would use for behavioral control are quite invasive […] you don’t want to put people on medication that could potentially make them wobbly or more unwell, or so forth and then create falls and infections and so forth.*

### 3.4. Providing Geriatric Knowledge to Nursing Home Staff

The second theme reflects stakeholders’ perceptions that, to better identify and manage comorbid conditions among nursing home residents with dementia, nursing home staff would benefit from additional training in geriatric care beyond that provided through higher education. Interviewed nurses reported not having completed geriatric-specific courses or placements, while care assistants and well-being companions reported lacking formal health-science education. Consequently, stakeholders reported that recruitment for care assistants and well-being companions prioritized personal attributes, such as a caring demeanor and creativity, over geriatric knowledge. For example, when asked about their qualifications and previous work-related roles, a well-being companion reported *I have never worked in a nursing home or care home before… I used to work in retail, and then obviously I had children and I was fortunate that I was able to be a stay-at-home mum. So it’s when my youngest [child] became sort of an age where I felt happy to leave her for a couple of hours after school, that I applied for this job. The job center said it’s perfect for you. You brought up children. You’re organizing children and play times and play groups for them because it’s very, very similar.* Lack of specific dementia knowledge among care assistants means that although they can access residents’ medical folders to know which diagnosed conditions they have, it is unlikely that they know what specific comorbid conditions involve and the treatment they require. Related to this, the GP stated: *I would say that even though they [care assistants and nurses] obviously do understand and they usually know those residents who have dementia they probably don’t always realize how extensive the effects of the dementia can be and also how certain symptoms might indeed relate. For example, I was talking about the sort of delirium aspect of dementia and we know when residents get relocated or come from hospital back to us and then nurses or care assistants, they might confuse that with more acute signs of infection.* The nursing home manager also acknowledged that, as the investigated nursing home is not a dementia-specific nursing home, in their opinion many staff members lack clinical knowledge to optimally identify and manage health issues in residents with dementia. To mitigate this, the manager decided to undertake a master’s degree in healthcare and has led workshops to staff. On this point, the nursing home manager said: *I did a workshop concerning geriatric care, where I talked to my staff about different types of conditions that elderly people might have and how that might impact how they are every day. For many staff, they didn’t know [about geriatric care] and they found this very helpful.* Finally, although the nursing home provides mandatory annual training to staff about dementia care, stakeholders explained that most courses are delivered online and remain unchanged year to year, missing the opportunity to impart to staff important clinical notions. Indeed, a well-being companion said: *It’s the same course year after year after year. It’s just a refresher, you do it that first time, then each year it’s just to refresh you, but you know exactly what’s coming up.*

### 3.5. Providing Services and Activities That Are Inclusive Towards Residents with Dementia and Other Comorbidities

According to nursing home staff, from Monday to Friday, residents with dementia are invited to participate in group activities together with residents without dementia. Because the proposed activities are not specifically designed for individuals with severe cognitive impairment, once cognitive decline becomes very severe, residents with dementia gradually withdraw themselves and decline participation to group-based activities. However, some staff members and informal caregivers recognized that lack of social interactions and stimulation among people with more advanced dementia may have implications for comorbid mood issues such as depression. For example, an informal caregiver said: *It’s still a very good home. I have no doubts about the intentions [of care assistants], but the delivery in terms of [referring to the relative with dementia] mood, I do wonder about that. They [staff members] offer things, but if [relative with dementia] is not awake, [relative with dementia] won’t get to them.* To offer an alternative form of social engagement, the nursing home team comprises two well-being companions dedicated to the delivery of one-to-one activities. Even though well-being companions expressed satisfaction with the level of engagement they believed providing to residents with more advanced dementia and other comorbidities, according to an informal caregiver, two well-being companions may be insufficient to meet all residents’ social needs and hence to prevent depressive symptoms.

### 3.6. Interacting with and Compensating for an Under-Sourced Healthcare System

This theme describes the challenges nursing home staff reported facing when interacting with external healthcare professionals. Participants reported uncertainty about which services or specialists to contact and long waiting times. The nursing home manager described a case in which multiple services declined to attend a resident with severe mental health needs: *So I was trying to look for any other people [specialists] that could come in and help us with the crisis that the resident was facing. It turned out that actually we have to deal with that crisis ourselves because they couldn’t come [mental health specialists] because they don’t. I think sometimes the barriers as well that when you work for the nursing home, there’s an expectation that you have nurses and they can provide all the care; whereas I think that expertise from community mental health practices would be very much important.* The GP commissioned by the investigated nursing home also reinforced how difficult it is to involve external specialists in the process of identifying or managing additional health conditions among residents with dementia: *It is very tricky to get them [residents] seen at home and even obviously getting referrals out to the hospital that can sometimes take several months.* Staff also highlighted shortages of community mental health specialists, resulting in long waits for residents. Consequently, staff reported that residents with severe mental health needs, including those displaying aggressive behavior, sometimes remain in the nursing home rather than being admitted to specialist services. As explained by stakeholders, this has negative consequences not only for residents’ own health, but also for the safety of other residents. For example, the nursing home manager said: *We continuously have to be the best advocate for residents, […] I think in [area where the nursing home is located], we’ve only been given two clinical specialist committees, mental health supports nursing homes where we know that we have so many residents in every care home that need that level of support. I mean when we’re waiting because there’s that waiting time only what we can do really is to have regular dialogue with GP.* Participants further noted that some services, such as telephone-based assessments, are poorly suited to medically assess people with dementia due to communication difficulties and often comorbid hearing issues. In this regard the nursing home manager said *I think you [referring to doctors] can’t assess residents over the phone basically because not only they [residents] can’t see you, they might not be able to trust you because they might get confused as well.* To address perceived shortcomings in the healthcare system, the nursing home established agreements with healthcare professionals, including a GP practice that provides weekly three-hour on-site visits and ongoing telephone and email support. According to stakeholders (e.g., one of the owners of the nursing home, the manager, and the GP), this collaboration enables timely identification and management of residents’ comorbid health issues and helps prevent more complex cases. Finally, stakeholders reported that the nursing home offered on-site physiotherapy services to minimize unnecessary travel for residents with dementia and comorbid mobility impairments.

### 3.7. Being Aware of One’s Responsibility Towards the Health and Wellbeing of Residents with Dementia

Participants highlighted the substantial responsibilities of their role, which some found overwhelming, while also clarifying the limits of their responsibilities. Care assistants pointed out that while they are responsible for the hygiene of residents, they are neither trained nor required to handle medical-related issues. For example, referring to the recognition of new health issues such as the suspect of urinary infection in a resident, a care assistant said: *We’ll let the nurse know because we don’t deal with these things.* So, when care assistants suspect a new health issue in a resident with dementia, the protocol is to report their concerns to the nurses. Similarly, nurses emphasized their daily medical responsibilities but noted that medication reviews for residents are conducted by the GP. At the same time, the GP emphasized the pivotal role nurses could/can play in identifying new health issues, highlighting the importance of strong clinical and geriatric training for nursing staff. For example, nurses prepare a weekly list of residents to be seen by the GP. According to the GP, this system has some flaws as it may result in less experienced nurses overlooking medical issues, while less confident nurses may request unnecessary GP visits, potentially leading to suboptimal use of GP time.

Participants highlighted adherence to regulations as essential for resident well-being and staff protection. For example, to safeguard the nursing home and its staff from cases of unexpected deaths, each resident is seen by the GP at least every two weeks, and their medications are reviewed every six months. In this respect, the owner of the nursing home said: *I believe that all our residents are required to see the GP every two weeks. […] In terms of sort of the unexpected death, if a doctor hasn’t seen somebody who’s died within two weeks that can potentially add additional complications and require coroner investigations and things like that.* These fears, combined with the demands of nursing and care work, can create a sense of burden among nursing and care staff. Indeed, despite being satisfied with their work environment and proud of their roles, some care assistants described the challenges of balancing residents’ needs, informal caregivers’ expectations, and their own emotional well-being. For example, a care assistant said: *Residents, family, you know everything, our health as well. It’s not easy to balance everything together, to be honest*. The nursing home manager also recognized these challenges and reported providing both group and individual emotional support, while remaining aware of the limits imposed by her own responsibilities and constraints. Concerning this, the nursing home manager stated: *We need to find time to be able to be selfish towards ourselves and find a time to really offload. […] But also, being able to understand that there will be situations that I might not be able to resolve. I’m very much aware of the barriers of circumstances that sometimes I cannot overcome. So just being honest and transparent with yourself, I think and then finding, where are your limits and where you need to seek support and resources certainly helps.*

Finally, informal caregivers also discussed their responsibilities. While they reported that transitioning a person with dementia to a nursing home often reduced their involvement in day-to-day decisions including treatment or medical decisions, many continued to feel responsible for aspects of residents’ care. However, the extent of this involvement varied according to personal preferences.

### 3.8. Second Study Aim

The following four themes addressed factors that facilitate identification and management of comorbidities in nursing home residents with dementia: delivering person-centred and relationship-based care; working as a coordinated team; welcoming criticism and striving for improvement; and fostering well-being among staff.

### 3.9. Delivering Person-Centred and Relationship-Based Care

This theme comprises four subthemes: undertaking a holistic approach of care; providing personalized care; investing in psychosocial care; and securing the trust of residents.

**Undertaking a Holistic Approach of Care**

In this subtheme, stakeholders reported that, when dealing with comorbidities in residents with dementia, a holistic approach is essential as physical and psychological care are interlinked. Care assistants reported how taking care of the psychological well-being of residents facilitates hygiene-related tasks and more positive behaviors during medical visits. Moreover, stakeholders (e.g., nurses, well-being professionals, the nursing home manager, the GP) explained how in residents with dementia psychological issues, such as agitation, are often the sign of physical discomfort arising from new health issues that cannot be verbalized. Additionally, taking care of residents’ families (e.g., communicating updates on the health of the resident) was considered part of a holistic approach. In this context, the owner of the nursing home said: *The reality of the fact that the care that we provide in the most sort of holistic sense incorporates the family.*

**Investing in Psychosocial Care**

This subtheme highlights the importance of providing psychosocial activities and of prioritizing psychosocial strategies when managing anxiety, agitation, and depression. Stakeholders reported that residents are offered daily group and one-to-one activities, and sometimes even pet therapy. While many residents initially require encouragement to participate, well-being companions reported that they generally enjoy them. A possible issue related to group activities delivered by well-being companions is that stakeholders reported delivering them to all residents; hence they may not be tailored for those with dementia. Residents with dementia can however benefit from one-to-one interactions with well-being companions. According to staff members, they can choose to be taken out in the garden, listen to music, videocall their family, and so on. In this regard, a well-being companion said: *They [referring to residents] might say to me, or can you come and help me this afternoon to write a letter? Or can I help with an e-mail or a diary, see whether I’ve got appointments to go out on with. I can go with them. I can sit in their rooms. I can take in puzzles or games or coloring. We can watch TV together. If they’re able bodied, I can take them out in the garden.* Physically mobile residents can be escorted to retail stores to buy personal items and can even go to the pub. Residents, including those with more advanced dementia, can also benefit from one-to-one chaplain visits. Indeed, in addition to leading a religious service once a month, the chaplain also reported providing one-to-one companionship through activities such as personal conversations, singing hymns with residents, and praying. The chaplain reported primarily interacting with residents who welcome their visits or whom nursing staff considered would particularly benefit from their support on a given day. In this regard the chaplain said: *there are often people who need my time more because of how they are and where they’re at, and the nurses sometimes say to me, could you call and see so, so today they’re a bit low or whatever.* Nonetheless, the chaplain recognized that having proper conversations may not always be possible with residents with more advanced dementia, especially when they have comorbid hearing problems: *Some who have dementia have serious hearing problems and so it makes it quite difficult to have a two-way communication.* Overall, according to stakeholders, residents are offered a range of activities tailored to their cognitive and physical abilities and personal preferences. Informal caregivers appeared grateful about this. For example, an informal caregiver said: *She [the resident] surprised all of us last year. She likes music, and she was at a residence event and she used her body in her wheelchair to actually move to this Hispanic music and she was moving her arms and so on […] and then another occasion she was just dancing on her two feet in a lounge with a male caregiver, and then he finished dancing with her and she went over to another caregiver who was sitting in a chair and she put her hands out like this and she got the care assistant up to dance with her, just very gently.*

Nursing home staff reported always using psychological interventions as the first attempt to deal with episodes of distress, anxiety, and depression; whereas using medications as last resort. Indeed, a nurse said: *Medication would be the last resort because we [nursing home staff] need to try and use everything that we have and to persuade and to reassure them [referring to residents] … if everything fails then only we give medications that’s prescribed.* Staff reported using several strategies to calm residents during psychological distress, including holding their hands, gentle touch, watching TV together, sharing a cup of tea, talking calmly, and taking walks. A well-being companion said: *It’s all about distraction and obviously if they’re still getting quite agitated, then obviously for their mental state, I would then take them from the room. But a lot of the times, 9 times out of 10, a little bit of distraction, a little bit of support […] I could have 25 people in the room if I can see someone is upset or distressed or getting anxious regardless of what’s going on, and the amount of people in there, I will make a point of going over to them, holding their hand and just being there for them and just helping to calm them. Then I will sit and spend more one-on-one time with them assisting them with whatever it is that they’re trying to do.* Overall, according to nursing home staff, being there for the residents through their preferred activities seems to work in reducing residents’ distress.

**Providing Personalized Care**

When people have multiple conditions, personalized care is especially important. In this subtheme, stakeholders emphasized the importance of adjusting their practices based on individuals’ needs and of involving residents in decision making. Both one of the owners of the nursing home and staff members provided examples of adjustments including residents’ physical environment, the type of activities proposed to residents, and ways of interacting with residents. For instance, to prevent confusion, residents with dementia may bring their own furniture when moving into the nursing home. A well-being companion said: *There’s a lot of rooms that have got people’s own furniture in them, which must be really comforting.* Another example, to communicate with residents with comorbid hearing impairments, electronic devices (e.g., tablets) are used. The nursing home manager said: *We’ve got one [resident] that is completely deaf and she’s using a special app with the tablet.* A further example, if a resident with dementia refuses to undertake a scheduled medical visit because they forgot about it or they do not understand the role of the doctor, care assistants reported rescheduling it. Staff, and especially care assistants and well-being companions, valued their flexibility in meeting participants’ needs. Nursing home staff reported that those residents with dementia who can still understand questions are encouraged to decide what and where to eat; whether they want to engage in group or one-to-one activity sessions; whether they want to undertake medical visits in the nursing home or elsewhere; and to communicate their wishes regarding end of life. In this regard, a care assistant said: *So the people [doctors] come here [at the nursing home] and they do eye tests for the residents. They come quite a lot, but some of the residents prefer to go to their own places [doctors/clinics] where they used to go before, especially the ones that got the capacity.* The GP said: *Those who have earlier onset of dementia for example who can still sort of communicate their wishes […] you can obviously discuss that [planning end-of-life treatments] a little bit more*. Involving residents in different types of decisions, including medical decisions, has been reported as an example of providing personalized care.

**Securing the Trust of Residents**

Staff valued securing the trust of residents because it facilitated hygiene-related tasks (including management of medications), social participation, and enabled medical visits. Some staff members explained the strategies they use to gain the trust of residents with dementia and comorbidities. For example, a well-being companion reported: *It’s just things like reading some poetry if they’ve got a book generally through things around their rooms because, you know they have lots of their own personal effects in that. So I’ll ask about photographs on the walls […].* Similarly, a care assistant said: *So we garden, sit there, I take pictures or try to film the garden and show it to some of the residents because they can’t go there. So I’ll try and bring the garden to them and [...] they will enjoy it.* Informal caregivers also emphasized the relationships between staff and their family members residing in the nursing home. For example, an informal caregiver said: *The staff are themselves. And that’s important because that’s how you can build a relationship with residents.* However, some stakeholders also reported that securing the trust of residents can be challenging during the first weeks after admission, especially when residents’ families find it hard to detach from the resident. For example, one of the owners of the nursing home mentioned a case where *A relatively new resident here has had family who have been, let’s say, so attentive that it’s got to the point where we feel like even after a month, we haven’t really been given a chance to win this gentleman’s trust or to kind of form those relationships.*

### 3.10. Working as a Coordinated Team

This theme comprises two subthemes: ensuring continuity of care and effective interdisciplinary collaboration.

**Ensuring Continuity of Care**

Stakeholders reflected on how continuity of care is key for timely identification of new comorbid health issues, and for effective management and treatment of ongoing conditions. About this, the nursing home manager said: *I think the level of care 24/7 support that we see those residents every single day certainly helps to identify when something is not right.* In the investigated nursing home, continuity of care was reported at several levels. Stakeholders reported that residents with dementia are checked hourly and cared for by a consistent team of care assistants and nurses, enabling staff to recognize behavioral changes that may indicate underlying health issues. In this regard, a nurse said: *We have hourly cheques in place. So our staff members are always vigilant and […] they maintain a strict documentation.* Residents requiring medical investigation are seen by a familiar GP, while all residents receive a GP visit every two weeks and medication reviews by a GP or community pharmacist every six months. Stakeholders also highlighted that continuity of care was maintained by adapting care to residents’ evolving needs. As part of a larger complex offering levels of care ranging from independent living to palliative and end-of-life care, nursing home staff felt they could often meet residents’ changing needs without requiring transfers or hospitalization. Corroborating this, the informal caregiver of a resident with dementia and other comorbidities reported that this continuity of care helped prevent hospitalization and related complications for their relative during a serious bowel issue. More specifically, the informal caregiver said: *She managed independent living, but then she had an acute bowel upset. [...] Certainly the one thing none of us wanted was our relative to go into hospital because we would not have been able to visit her, with her dementia that would have been really very difficult. […]. She couldn’t manage in the independent living but there wasn’t an acute bed in the nursing home and thankfully, the [owners of the nursing home] helped us find her a room and they were able to nurse her medical needs, which was quite difficult when you’ve got someone with dementia who doesn’t know when to go to the lavatory but needs to go to the lavatory, doesn’t remember to call somebody to help her. […] The good news is she got a lot better. She got over her acute bowel situation and things settled down again, but it was abundantly obvious that she couldn’t go back into independent living, and she’d really probably been living on a tightrope anyway, so that was just the final straw. And that’s when she became a nursing home resident.*

**Effective Interdisciplinary Collaboration**

This subtheme was reported by staff as the main reason why they can successfully meet the complex needs of residents with dementia and comorbidities. Staff discussed communication with residents’ GP and other specialists, with other nursing home staff members, and with residents’ informal caregivers.

Stakeholders documented coordination of care and exchange of medical information between specialists. On those occasions where external specialists (e.g., mental health specialists) are involved in the care of residents, the residents’ GP is updated via email. In this regard, the GP said: *I usually expect to get about 4–5 emails a day about various things. I mean, sometimes it’s just about a person’s medication or so, but they [nurses] will e-mail me during the week about more acute things, like if they suspect that somebody might have a urine infection and those sorts of things. Of course, if they have much more acute things, then they get in touch. They usually ring the surgery and get hold of me. Or if I’m not at the surgery, then they’ll obviously speak to whoever is on duty on that day.*

The chaplain also reported having been contacted by nurses to calm down some residents with dementia who showed anxiety: *We have a resident who can be very anxious and sometimes if she’s very anxious, I think they have phoned me up in the past and asked me to come in and see them.* Participants also described the oral and written communication that occurs between nursing home staff. Staff meet each morning to discuss current medical issues, conduct handovers between shifts, and maintain written records of hygiene-related tasks and administered medications. When facing unusual health issues, the manager, who is in the middle of residents’ rooms, reported being always available to be consulted. In this regard, the nursing home manager stated: *So there’s two handovers basically morning with night people and the day nurses and same in the evening the day staff is giving her hand over to the night staff. So I think that communication is very, very important. I cannot see the situation that you’re coming in on the floor after a few days off and then just going straight to work. You know, things might change, isn’t it? Someone might be on the way or, you know, there is a significant event.*

Finally, participants shared accounts of interactions between nursing home staff and residents’ informal caregivers. Nursing home staff notify informal caregivers every time the resident has a major visit, is subject to a medication review, and is suddenly hospitalized, but also to inform about minor daily updates (i.e., whether their family member refused to bath on a specific day). Annual reviews also occur where residents, their informal caregivers, and their main care and nursing staff meet to brief the informal caregiver on the health state of the resident. Informal caregivers also reported examples of situations where they brough up complaints to the nursing home staff. An informal caregiver reported an example where their complaint was met: *I did have to make a complaint once to the manager and it was acted through and eventually not long after that person left.* Another informal caregiver reported their conflict between wanting to make a complaint to prevent falls in their relative with dementia but at the same time not wanting to deteriorate their relationship with nursing home staff: *I was just thinking all I’m doing is speaking up for her, but I didn’t want. I’m not a rowdy, argumentative person. I didn’t go in, you know, blazing and so on. And I didn’t feel satisfied. And I just thought, I don’t know what else to do because it’s a very delicate thing. You’re paying for this and there is a contract and they are meant to be doing certain things, but of course it’s my mother who’s the subject. I’m giving them feedback and it’s an awkward relationship at times and a difficult one, quite a nuanced one […] I have power of attorney and so I can make decisions. I’m the one that pays for everything. So this whole thing about when there are complaints, you want to be in relationship. I want to be in relationship with these people. I don’t want to build up a negative profile.* Overall, communication between staff and families is something that some stakeholders perceived as difficult but highly important.

### 3.11. Welcoming Criticism and Striving for Improvement

This theme refers to all the strategies and innovations nursing home staff reported adopting to foster improvement of care and of its environment in response to emerging needs and new knowledge. For example, in relation to emerging knowledge on dementia care, a nurse stated: *We’ve done a lot of adjustments... as you know, with dementia training, they say things with color on the people with dementia. So some of our cutleries is colorful, the door toilet stickers are yellow or green. So we try to incorporate all the colors that we’ve been taught that attract people with dementia that they can see. We’ve incorporated different cutlery, different cups, uh different activities that suit every level of dementia. I feel like with us, we are getting there. We are on our we are on track. We’re running and anything else that we can learn that’s new, we embrace it.* In relation to adapting the environment according to the needs of residents with dementia and comorbidities, an informal caregiver shared the following example to highlight that this intention is not always met: *As my mother’s condition developed, I became more and more concerned and hit my head against a brick wall about will you do something about making sure that this door cannot be opened by my mother because she’d had this horrible fall and I thought, what if she does? It’s very narrow, winding. You’ve got the picture. I should think, I thought just one day in her head, whatever’s going on, she decides to go down there and she breaks her neck. That was my worst-case scenario and I just thought this is going to be really bad for a lot of reasons. I was also thinking about [the nursing home], and I just bashed my head against a brick wall. Eventually they decided to put a gate in. Thank goodness. But even then, on weekends, I noticed I come, and it wasn’t always closed.* However, nursing home staff reported welcoming criticism. For example, the nursing home manager explained that they have quality improvement weeks where staff and residents can identify areas of improvement. Moreover, when an avoidable health issue occurs in a resident, the manager reported organizing an event where the “lessons learned” from that specific case are discussed. Additionally, the manager put an anonymous suggestion box that allows informal caregivers to report concerns.

### 3.12. Fostering Well-Being Among Staff

Promoting well-being among staff was perceived, especially by the manager and one of the nursing home owners, as something indispensable to keep staff turnover low and, consequently, to ensure continuity of care. Continuity of care was reported as being indispensable as familiarity with residents facilitates early identification of new health issues and the identification of treatments that do not interfere with residents’ conditions. Staff (i.e., nurses, care assistants, well-being companions) expressed gratitude to work for owners that care about their well-being. A care assistant said: *It doesn’t feel like we are in an agency now. It’s just like a family.* Staff also reported benefitting from emotional support through discussions with the nursing home manager and other colleagues, which facilitated the sharing of advice on residents’ health and well-being. About this a care assistant said: *Our duty manager, we can talk with her about everything. She’s open mind. And every time you have a problem, or you have an issue, you can talk to her.*

### 3.13. Conceptual Model

Figure one presents the conceptual model developed from the thematic analysis, illustrating stakeholders’ perceptions of the factors influencing the identification and management of comorbidities in nursing home residents with dementia. The model is organized across four interconnected domains. At the broadest level, the wider healthcare context, particularly the perceived under-resourcing of the healthcare system, was viewed as shaping the context in which care is delivered. Within the nursing home, organizational factors, including working as a coordinated team, welcoming criticism and striving for improvement, and fostering well-being among staff, were perceived to facilitate the identification and management of comorbidities. Stakeholders also highlighted the importance of staff capability and care practices, including geriatric knowledge, responsibility, delivering person-centred and relationship-based care, and providing services that are inclusive towards residents with dementia and other comorbidities. Finally, resident-related challenges, particularly the consequences of dementia, were perceived as increasing the complexity of recognizing and managing comorbidities. Rather than depicting causal relationships, the model synthesizes how stakeholders perceived these contextual, organizational, staff, and resident-related factors to interact in shaping the identification and management of comorbidities in residents with dementia.

## 4. Discussion

This single-site qualitative interview study explored the perspectives of fifteen stakeholders from a well-resourced UK nursing home regarding the challenges and facilitating factors associated with identifying and managing comorbidities in residents with dementia. The findings suggest that these factors operate at multiple, interconnected levels, encompassing the wider healthcare context, organizational factors within the nursing home, staff capability and care practices, and resident-related challenges. The following discussion considers each of these domains in turn.

Starting from the first domain, stakeholders perceived the wider UK healthcare context as under-resourced and described how this suboptimal context challenges prompt identification and management of comorbidities in residents with dementia. A previous UK expert consensus study also reported limited access to GPs and other external specialists as an important barrier to identification and management of comorbidities among nursing home residents with dementia [25]. However, as outlined in the next paragraphs, in this single-site qualitative interview study, staff explained how they adapted to these perceived limitations of the wider healthcare context (e.g., through a commissioned GP) to promptly address the healthcare needs of residents with dementia.

Moving to the second domain concerning organizational factors within the nursing home, working as a coordinated team, fostering well-being among staff, and welcoming criticism and striving for improvement, were all perceived as facilitators in the identification and management of comorbidities in residents with dementia. Importantly, continuity of care was perceived as a factor that facilitates both prompt identification of new health issues and management of existing comorbidities. However, ensuring continuity of care in nursing homes can be challenging [36] due to high staff turnover (i.e., 34% resigning in one year) as newly hired staff are not familiar with residents’ needs [37]. In the investigated nursing home, the owner reported that currently employed staff had been working an overage of 6.2 years. Interviewed staff also expressed their intention to continue working in the investigated nursing home. Although this single-case study was based on a well-sourced nursing home, reflecting on what made staff working and wanting to continue to work in the same nursing home for several years may be exemplary for other UK nursing homes. First, all investigated care assistants, nurses, and well-being specialists reported appreciating the opportunity to have one-to-one consultations and well-being chats with the nursing home manager. Second, staff (e.g., care assistants) explained always attending the same residents, which facilitates cohesion. Third, care assistants and well-being companions were grateful for the freedom they had while undertaking daily tasks. Finally, nursing and care staff expressed mutual support. Overall, staff members described how the nursing home provided a range of benefits that, according to them, were not present in their previous workplaces.

Another element that was perceived as facilitating continuity of care in the investigated nursing home is the employment of a GP. According to one of the nursing home owners, the nursing home manager, and nursing and care staff, the GP played a key role by visiting each resident at least every two weeks, providing remote support to the nursing team, and coordinating care with external specialists. For example, stakeholders reported that online interactions between the GP and other specialists allowed the GP to provide residents with solutions while they waited to be visited by external specialists. Having residents seen regularly by a GP also decreased reliance on nurses who, according to the judgment of the manager and GP, had limited clinical and geriatric knowledge. Indeed, previous research has identified the need for additional geriatric and dementia-specific education among UK nurses working in residential care [38], and this is partly due to UK nursing degrees not including mandatory clinical placements in residential or dementia care settings. Moreover, the commissioned GP arrangement enabled residents to benefit from regular in-person medical visits. This is important as nowadays, some GPs and other specialists propose virtual communication but, as stakeholders have highlighted in this study, these are inappropriate for people with dementia, especially for those who have comorbid hearing difficulties and/or confusion [39].

Overall, the commissioned GP arrangement enabled the investigated nursing home to compensate for some of the perceived limitations of the wider healthcare system, including limited geriatric expertise among nursing staff and increasing waiting times for in-person specialist care [40,41]. However, this arrangement relied on additional financial resources and may not be affordable or feasible for many nursing homes. Consequently, while this single-site qualitative interview study illustrates the potential value of consistent GP involvement in supporting continuity of care for residents with dementia and comorbidities, it should not be interpreted as evidence that commissioning additional GP services represents a universally applicable solution. Instead, these findings highlight the importance of ensuring continuity, accessibility, and effective multidisciplinary collaboration within primary care, regardless of the specific service model adopted. Future research should compare different models of primary care provision across nursing homes to identify approaches that are both clinically effective and economically sustainable.

While communication among staff members was generally perceived as effective and positive, interactions between staff and residents’ informal caregivers were, on some occasions, perceived as challenging by both staff and informal caregivers. Aligned with previous evidence [42], informal caregivers were grateful for the updates they receive from staff regarding residents’ health, for the possibility to interact with the GP directly, and for the annual reviews where they can discuss in depth the health of the residents with the care team. However, in terms of treatments, informal caregivers were finding it hard to understand what the limit is between what they should delegate to the nursing home and what they should decide on. Informal caregivers also appeared hesitant about the possibility of expressing complaints to staff members as they were afraid of undermining a much-valued relationship. Similarly, nursing home staff also reported some contrasting feelings regarding interaction with residents’ families. Staff (e.g., nurses) viewed family involvement (e.g., communication of health episodes to residents’ families) as essential to holistic care but also time-consuming and challenging, partly due to the emotional adjustment families face when placing a relative in a nursing home [43]. Although not explicitly suggested by participants, the findings indicate a need for additional support to facilitate communication and shared decision-making between nursing home staff and residents’ informal caregivers. This role could potentially be fulfilled by a dedicated professional, such as a psychologist or another specialist with expertise in dementia care, who could provide education, support communication, and help families navigate treatment decisions. Whether such a role is feasible, acceptable, and cost-effective within UK nursing homes requires further investigation.

Moving to the third domain, stakeholders highlighted the importance of staff capability and care practices, including increasing geriatric knowledge among nursing and care staff, being aware of one’s responsibilities, delivering person-centred and relationship-based care, and providing services that strive to be inclusive towards residents with dementia and other comorbidities. First, the importance of increasing geriatric knowledge among nursing and care staff has already been mentioned in the second domain. However, it must be noted here that the investigated nursing home is not a dementia-specific nursing home and therefore it is not mandatory for its staff to have received formal training on dementia. Provisions of more specific geriatric courses and training in nursing curricula may equip all nurses with the specific geriatric knowledge and confidence that is necessary to identify health issues in residents with limited communication [44,45,46]. Moreover, in this case study, according to stakeholders’ accounts, greater knowledge and confidence among nurses would have facilitated a more efficient way of working with the GP, for the benefit of the residents. In this regard, a review study reported some effective initiatives including requiring nurses to submit dementia specific portfolios each year evidencing education in their fields to the Nursing and Midwifery Council [47].

Second, staff awareness of their responsibilities and of the boundaries of their roles could be interpreted as an additional element of good teamwork and subdivision of roles. At the same time, however, some staff members expressed an overwhelming feeling related to awareness of their responsibilities. In this case study, the nursing home manager acted as direct source of emotional support for nurses, care assistants, and well-being specialists, but not all nursing homes may benefit from a supportive manager. Second, about delivering person-centred and relationship-based care, the importance of undertaking a holistic approach and of providing personalized care have been widely discussed as essential elements of good dementia care in general [48,49]. The need to invest in psychosocial care and, when possible, to prioritize it over medication has also been supported by evidence on deprescribing in residential care [50,51,52].

The last domain concerning resident-related challenges focuses on the difficulties of identifying and managing comorbidities while dealing with the symptoms of dementia. Indeed, when asked which comorbid conditions were most difficult to manage in residents with dementia, staff did not mention specific health conditions but, instead, indicated that the greatest challenge was identifying and treating health conditions in residents with advanced dementia and who are, therefore, unable to effectively communicate and unaware of their health problems. Moreover, among dementia-related symptoms, staff reported frequently dealing with depression, anxiety, and agitation, and described how these behavioral issues can obstacle optimal identification and management of comorbidities (e.g., medical visits may have to be rescheduled due to residents being agitated). Some care assistants and well-being companions felt confident managing these symptoms but acknowledged that their strategies were based on intuition rather than scientific knowledge. For example, the well-being team and care assistants felt utilizing touch helped soothe many residents from agitation, however there was little certainty in knowledge. Researchers have widely reported the benefits of therapeutic touch in soothing comorbidities such as sleeping disorders [53], but greater effort should be put to ensure research-led recommendations reach nursing homes in a way that staff can act with a greater degree of confidence. Overall, consistent with the previous domains, stakeholders suggested that providing additional clinical, geriatric, and dementia-specific training to care assistants, well-being companions, and nurses could improve their ability to deliver evidence-based psychosocial activities for residents with dementia and comorbidities [54].

This study has several limitations. First, on a couple of occasions, staff reported different ways of doing things. As we did not conduct an observational study, it is difficult to understand what the standard practice is. Second, the participating nursing home was the only nursing home who accepted to participate after having contacted 14 nursing homes, leading to a self-selection bias. Third, this is a single-site qualitative interview study based on a UK nursing home that has obtained several prizes for the quality of its care. It is therefore possible that the findings reflect a positive deviant case rather than a typical nursing home, consequently limiting transferability of results. Hence, this study may have undetected issues present in other nursing homes. Fourth, although participation was voluntary, some staff members may have felt implicit pressure to participate because the invitation to join the study was communicated by one of the nursing home owners. Moreover, the interviews were conducted during working hours. Although participants were informed that participation was voluntary and interviews were conducted in a private room, some staff may nevertheless have felt constrained when discussing challenges within their workplace and they may have presented themselves in a socially desirable way. This could partly explain the predominantly positive portrayal of the nursing home in the findings. Fifth, the informal caregiver sample was limited, comprising only two participants whose relatives had resided in the nursing home complex for several years. Consequently, their perspectives may not be representative of caregivers with more recent admissions. Sixth, although residents with advanced dementia have difficulties taking part in online interviews, a limitation of this study is that it did not include the voices of residents with dementia and comorbidities. Seventh, this study lacks from methodological triangulation. Eight, because participants commonly discussed dementia-related communication and behavioral challenges rather than specific comorbid diagnoses, some findings may reflect general dementia-care practices rather than challenges unique to comorbidity identification and management. Nonetheless, this is the first single-site qualitative interview study on the topic of comorbidities in UK nursing home residents with dementia. Additionally, the semi-structured nature of the interviews, their considerable length, and the eight different types of stakeholders that were interviewed allowed researchers to go in depth with the topics mentioned.

## 5. Conclusions

This single-site qualitative interview study offers context-specific insights into how stakeholders perceived the challenges and supports involved in identifying and managing additional conditions and health issues among residents with dementia. Stakeholders described these factors as operating across four interconnected domains: the wider healthcare context, nursing home organization, staff capability and care practices, and resident-related challenges. While dementia-related communication difficulties and perceived limitations of the wider healthcare system increased the complexity of identifying and managing comorbidities, organizational features such as coordinated multidisciplinary working, continuity of care, supportive leadership, and person-centred care were perceived to facilitate care of residents with dementia and other comorbidities. Rather than identifying a single solution, the findings suggest that participants perceived management of comorbidities in residents with dementia to be supported by coordinated action across multiple levels of the care system. Future research should examine the transferability of this conceptual model across different nursing home settings and evaluate organizational and healthcare service models that can support sustainable, person-centred management of comorbidities in residents with dementia.

## Figures and Tables

**Table 1:** Details on participants’ roles.

Participant	Role	Years Working in the Nursing Home	Years Working in the Care System	Gender	Professional Qualifications	Training in Dementia Care	Employment Status
1	Care assistant	2	5	Female	None	No formal training besides what provided in the nursing home	Employed
2	Care assistant	5	5	Female	Care certificate	No formal training besides what provided in the nursing home	Employed
3	Care assistant	1	2	Female	BSc in Civil engineering and MSc in environmental engineering	No formal training besides what provided in the nursing home	Employed
4	Care assistant	6	30	Female	None	Dementia course in level 3	Employed
5	Well-being companion	4	4	Female	None	No formal training besides what provided in the nursing home	Employed
6	Well-being companion	1	17	Female	None	No formal training besides what provided in the nursing home	Employed
7	Well-being companion	3	11	Female	None	No formal training besides what provided in the nursing home	Employed
8	Owner	20	20	Male	Undertaking a degree in Psychology at the time of data collection	No formal training on dementia	Employed
9	Manager	6	18	Female	Master’s in nursing	Adult social care leadership course level 5	Employed
10	Registered nurse	8	8	Female	Honours degree in Nursing	No formal training besides what provided in the nursing home	Employed
11	Informal caregiver	n/a	n/a	Female	NA	NA	Retired
12	General Practitioner	10	10	Male	Medical school	No specific training but accumulated experience at a local GP and during medical training	Employed
13	Informal caregiver	n/a	n/a	Female	NA	NA	Retired
14	Clinical lead nurse	9	16	Female	Nursing	No formal training besides what provided in the nursing home	Employed
15	Chaplain	6	6	Female	NA	NA	Employed

NA = Not Applicable.

## Data Availability

The anonymized data used for this study can be found in Open Science Framework at the following link https://osf.io/6wvtd/ (accessed on 1 August 2026). Reference: Sabatini, S. (9 October 2024). Identification and management of dementia comorbidities: case study in a UK nursing home. Retrieved from https://osf.io/6wvtd/.

## Supplementary Materials

The following supporting information can be downloaded at: https://www.mdpi.com/article/10.3390/healthcare14162601/s1, Supplementary Text S1: Interview questions for nursing home staff/professionals.
