# Supplementary material for: Stakeholder Perspectives on Identifying and Managing Comorbid Health Conditions Among Nursing Home Residents with Dementia: A UK Single-Site Qualitative Study
Source: Healthcare (Basel). 2026 Aug 18;14(16):2601. doi: 10.3390/healthcare14162601 (PMC13512145; doi:10.3390/healthcare14162601)
Supplement: Supplementary file 1 [file healthcare-14-02601-s001.zip › healthcare-4397768-supplementary.pdf]

Supplementary Text S1

Interview questions for nursing home staff/professionals

Thank you for taking part in this interview today. I am XXX and a postgraduate student in Psychology at the University of Surrey. In the call is also present XXX who is lecturer in Psychology at the University of Surrey. Her research focuses on several issues related to ageing, dementia, and caregiving. The final aim is to promote better quality of life in older people, including people with dementia, and their carers.

In this specific study we want to understand the perspective of different nursing home staff/professionals in dealing with comorbidity in people with dementia living in nursing home, as well as the perspective of carers of people with dementia who reside in the care home you work for. We envisage that the interview will last between 20 and 30 minutes. Before starting the interview, do you have any questions for me or XXX?

Also, before starting the interview I would like to ask for your permission to audio-record the interview. I will use this recording to transcribe our conversation. Once this is done, I will permanently delete the audio-recording. Moreover, the transcript of our conversation will be anonymised so no one can recognise you. So if you do not have any questions for me or Serena, I will start with the interview.

1. In your opinion, which comorbid conditions to dementia are most common in the nursing home you work for?
2. Which type of comorbid conditions to dementia you find most difficult to manage? Why?
3. In your opinion, what are the most salient issues associated with management of comorbidities in people with dementia?
4. Which plans/protocols (if any) are in place in the nursing home you work for to deal with comorbidities in people with dementia?

5. Which type of specialists are generally involved in the care of people with dementia and other comorbidities?
6. How much do these different specialists interact with each other's when addressing comorbid health issues in people with dementia?
7. What is currently done well in order to manage comorbidities in people with dementia in the nursing home you work for?
8. What could be done better to manage comorbidities in people with dementia in the nursing home you work for, as well as in other nursing homes?

We would like to thank you very much for your participation in this study. It has been incredibly insightful talking to you.

Interview questions for carers of people living with dementia in nursing homes

Thank you for taking part in this interview today. I am XXX and a postgraduate student in Psychology at the University of Surrey. In the call is also present XXX who is lecturer in Psychology at the University of Surrey. Her research focuses on several issues related to ageing, dementia, and caregiving. The final aim is to promote better quality of life in older people, including people with dementia, and their carers.

In this specific study we want to understand the perspective of carers of people with dementia and other comorbid conditions who reside in nursing home, as well as the perspectives of those who take care for your family member with dementia in the nursing home.

We envisage that the interview will last between 20 and 30 minutes. Before starting the interview, do you have any questions for me or XXX?

Also, before starting the interview I would like to ask for your permission to audio-record the interview. I will use this recording to transcribe our conversation. Once this is done, I will permanently delete the audio-recording. Moreover, the transcript of our

conversation will be anonymised so no one can recognise you. So if you do not have any questions for me or Serena, I will start with the interview.

1. Has your family member with dementia been diagnosed with one or more other comorbid health conditions before or after having moved to the nursing home?

   [please ask the following only to carers of those who have received a new diagnosis while being in nursing home]. I would like to ask you a few follow-up questions to better understand how the diagnostic process unraveled for your family member.
2. How many specialists were involved in the diagnostic process?
3. How long did it take to your family member to receive a diagnosis from first appearance of symptoms?
4. What, in your opinion, has been done well by staff/professionals working in the nursing home to identify the symptoms in your family member and help him/her obtain a diagnosis?
5. What could staff/professionals working in the nursing home have done better? [please ask the following questions only to carers of those who already had one or more comorbid diagnosis while being in nursing home]. I would like to ask you a few follow-up questions.
6. Have the comorbid conditions in your family member with dementia deteriorated since when moving to nursing home? If so, more or less than what expected for this/these condition/s?
7. How well is this/are these comorbid condition/s managed in your family member by nursing home staff/professionals?
8. Is there something they could have done better?

9. [if the carer mentions some issues, please ask follow-up questions] Why do you think they did not act this way? What are the biggest barriers? E.g., is it due to lack of training, lack of time to dedicate to each resident due to busy routines, etc?

We would like to thank you very much for your participation in this study. It has been incredibly insightful talking to you.
